# Autonomous motion and control of lower limb exoskeleton rehabilitation robot

**DOI:** 10.3389/fbioe.2023.1223831

**Published:** 2023-07-14

**Authors:** Xueshan Gao, Pengfei Zhang, Xuefeng Peng, Jianbo Zhao, Kaiyuan Liu, Mingda Miao, Peng Zhao, Dingji Luo, Yige Li

**Affiliations:** ^1^ School of Mechatronical Engineering, Beijing Institute of Technology, Beijing, China; ^2^ China Shipbuilding Industry Corporation, No.713 Institute, Zhengzhou, Henan, China

**Keywords:** improved adaptive particle swarm, admittance control, dual RBF adaptive sliding mode control, active control of lower limb exoskeleton, human lower limb rehabilitation frontiers

## Abstract

**Introduction:** The lower limb exoskeleton rehabilitation robot should perform gait planning based on the patient’s motor intention and training status and provide multimodal and robust control schemes in the control strategy to enhance patient participation.

**Methods:** This paper proposes an adaptive particle swarm optimization admittance control algorithm (APSOAC), which adaptively optimizes the weights and learning factors of the PSO algorithm to avoid the problem of particle swarm falling into local optimal points. The proposed improved adaptive particle swarm algorithm adjusts the stiffness and damping parameters of the admittance control online to reduce the interaction force between the patient and the robot and adaptively plans the patient’s desired gait profile. In addition, this study proposes a dual RBF neural network adaptive sliding mode controller (DRNNASMC) to track the gait profile, compensate for frictional forces and external perturbations generated in the human-robot interaction using the RBF network, calculate the required moments for each joint motor based on the lower limb exoskeleton dynamics model, and perform stability analysis based on the Lyapunov theory.

**Results and discussion:** Finally, the efficiency of the APSOAC and DRNNASMC algorithms is demonstrated by active and passive walking experiments with three healthy subjects, respectively.

## 1 Introduction

In recent years, the number of stroke patients has been increasing. More precisely, the statistics show that more than 4 million new stroke patients are diagnosed each year in Europe, the United States, and China (Zhang et al., 2017; Paraskevas, 2020). At the same time, the problem of motor dysfunction in patients due to stroke has significantly increased, while approximately 65% of these patients require rehabilitation (Miao et al., 2023). However, the traditional manual rehabilitation has many problems, such as strained medical staff and insufficient manpower to ensure consistency of repetitive training. Moreover, it can cause greater financial stress to patients (Gittler and Davis, 2018). The Lower Extremity Exoskeletal Rehabilitation Robot (LEERR) can drive the patient’s legs to perform repetitive rehabilitation exercises through active and passive training, in order to reshape its motor nerves and restore its independent walking ability (Xing and Lv, 2019; Bae et al., 2020). The above studies have important research implications for understanding the rehabilitation needs of stroke patients, improving traditional rehabilitation methods, and exploring the application of lower extremity exoskeletal rehabilitation robotics. These studies are expected to provide more effective and sustainable rehabilitation programs for stroke patients and improve their quality of life and rehabilitation outcomes.

One of the most critical issues of LEERR is the accurate identification of the human body’s motion intention, and the human-robot collaboration based on less human-robot interaction force for active rehabilitation (Chen et al., 2022). Force sensors, Inertial Measurement Unit (IMU), and Electromyography (EMG) sensors are usually used to capture the human motion intention (Meng et al., 2020). Xie et al. used EMG information combined with admittance control to perform active control of an upper limb exoskeleton robot for several tasks such as grasping and rehabilitation training (Xie et al., 2021). Huang et al. used force sensors as the basis for human-robot interaction, and generated motion reference trajectories using a conductive adaptive fuzzy algorithm, which provides a theoretical basis for the realization of human active rehabilitation movements (Huang et al., 2022). The IMU can measure the foot trajectory, and it is used to identify different terrain levels and thus decide on different movement patterns (Gao et al., 2020). Zhuang et al. used impedance control to capture the human motion intention through myoelectric signals and track it through PD control, in order to obtain satisfactory knee joint flexibility control results (Zhuang et al., 2020). In this study, the interactive force of the subject’s thigh and calf was measured by a two-dimensional force sensor to identify the user’s motion intention.

Admittance control (Chen et al., 2021) and impedance control (Soliman and Ugurlu, 2021) are more widely used in lower limb exoskeletons, due to the fact that they allow the motor to always move in the direction where the human-robot interaction force becomes smaller, ensuring flexibility between the human and the robot (Losey and O’Malley, 2017). In active patient rehabilitation training, the coupling and interaction between the human and the robot change at any time, and therefore the controller should be able to adjust the appropriate parameters according to these changes (Cousin et al., 2021; Wang et al., 2021; Guo et al., 2022). After the desired trajectory of the human body is planned in the outer loop of the control system using impedance/admittance control, the trajectory should be efficiently tracked by the inner loop controller to make the human-robot interaction force reach the user’s expectation (Rahmani and Rahman, 2020; Lee et al., 2021). With the development of artificial intelligence and computer technology, more researchers combined artificial intelligence techniques with traditional control techniques, and then controlled nonlinear systems. For instance, Babak Esmaeili et al. proposed an adaptive iterative learning integral terminal sliding mode controller. The obtained simulation results demonstrated that the latter overcomes the problem of perturbations and performed effective trajectory tracking (Esmaeili et al., 2022). There are many system variables in the exoskeleton control system that cannot be measured, and thus they should be approximated and compensated (Yang et al., 2021). Razzaghian designed a finite-time fractional-order non-singular fast terminal sliding mode control, and used a fuzzy neural network algorithm to approximate external perturbations, which ensures the exoskeleton control finite-time convergence and robustness (Razzaghian, 2022). Control system uncertainty and external perturbations are the main causes of control system instability. Several algorithms such as neural networks (Yang and Gao, 2019), machine learning (Sun et al., 2022), adaptive control (Wu et al., 2018; Sun et al., 2021), sliding mode control (Kong et al., 2019), and intelligent swarms, are mainly used to solve these problems (Soleimani Amiri et al., 2020; Guo et al., 2022; Zhang and Zhang, 2022).

Inspired by the study on lower limb exoskeletons, the main contributions of this paper are summarized as follows: This paper proposes an improved adaptive particle swarm optimization admittance control algorithm and a dual RBF neural network adaptive sliding mode controller for passive control of the lower limb exoskeleton robot. The proposed methods aim to achieve flexible control and accurate tracking of nonlinear and bounded perturbed systems during active and passive walking training, while reducing human-robot interaction force and energy consumption. The stability of the proposed controllers is verified using the Lyapunov theory.

## 2 LEERR system design

In this section, the system design process of LEERR is detailed, and three subsystems are designed: the mechanical structure of LEERR, the hardware control system, and the multi-source sensor information system. The structure of LEERR has seven degrees of freedom, where the hip and knee joints of both legs are active drive joints, the ankle joint is a passive degree of freedom, and the exoskeleton is mounted on the slide of the bracket to adjust the robot height. The LEERR hardware control system consists of robot controller, drive, and host computer units. The multi-source sensor information system provides the real-time force, current, and angle signals to the exoskeleton control system.

### 2.1 LEERR structure

**FIGURE 1:**
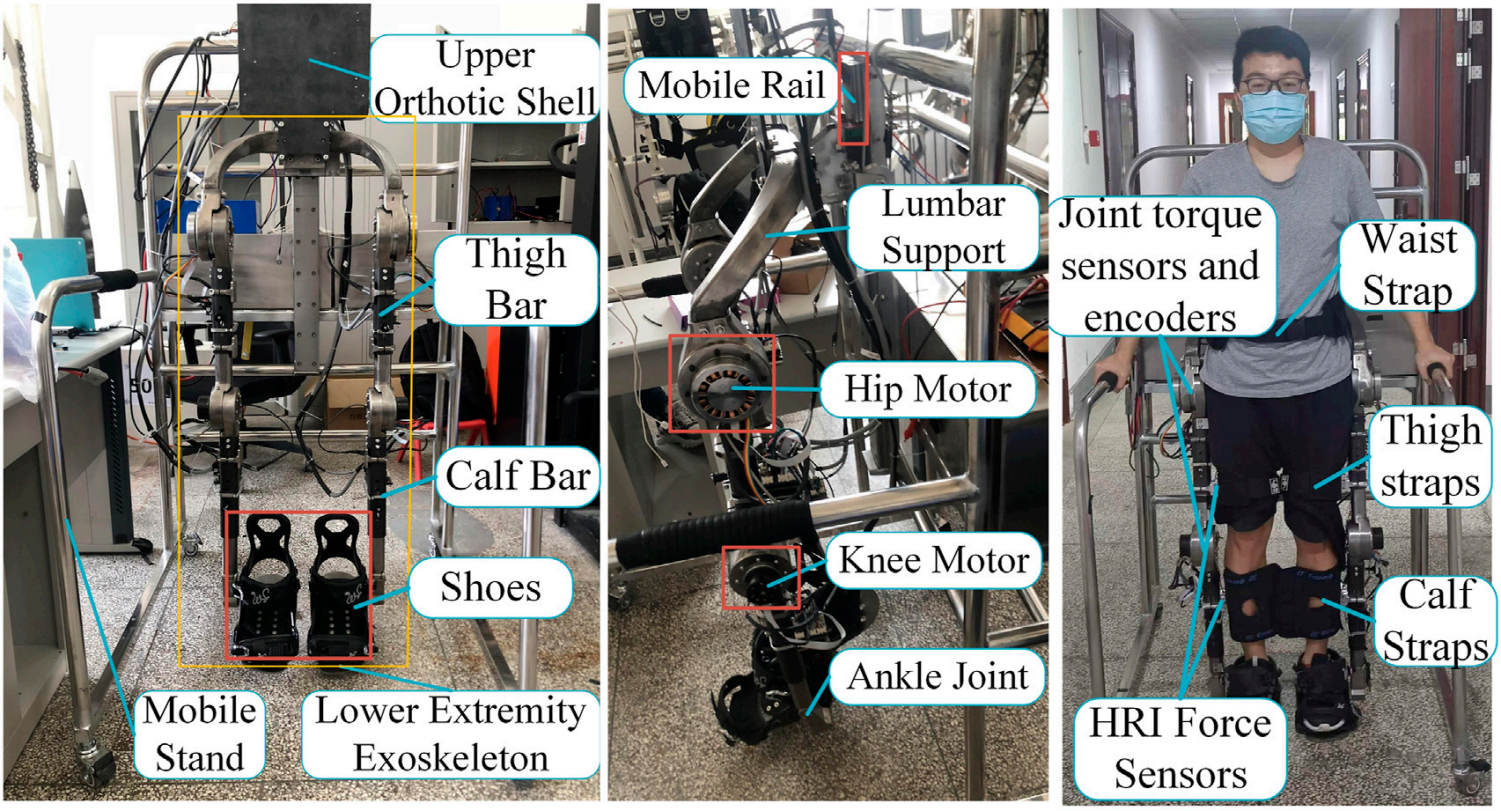
LEERR structure and installation position of the equipment.

The LEERR human-robot coupling model is shown in Figure 1. The structure of the lower limb exoskeleton robot includes the upper orthotic shell, mobile rail, thigh bar, calf bar, hip motor, knee motor, ankle joint, support shoe, straps, and four-wheeled mobile platform support. The four wheels of the mobile stand are non-powered, and its forward motion relies on the step of the lower limb exoskeleton to drive. The human body is connected to the waist, thighs, calves, and soles of the exoskeleton by straps. The lower limb exoskeleton is fixed on the slide rail of the mobile bracket so that the exoskeleton can float up and down with the body’s center of gravity to make the patient more comfortable during walking. The length of the thighs and calves of the exoskeleton and the width of the waist can be adjusted using pins. It is suitable for people having heights in the range of 155–195 cm and weights less than or equal to 110 kg. Table 1 shows the mechanical specifications of the exoskeleton, illustrating the rated torque, speed, and rotation angle range of the hip and knee motors.

**TABLE 1:** Mechanical motion properties of the lower limb exoskeleton.

Column 1	Hip joint	Knee joint
Rated torque	65 Nm	45 Nm
Rotational Speed	20 rpm	25 rpm
Range of motion	FE: −10°–25°	FE:0°–65°

Note: FE, Flexion/Extension.

### 2.2 LEERR hardware control system

**FIGURE 2:**
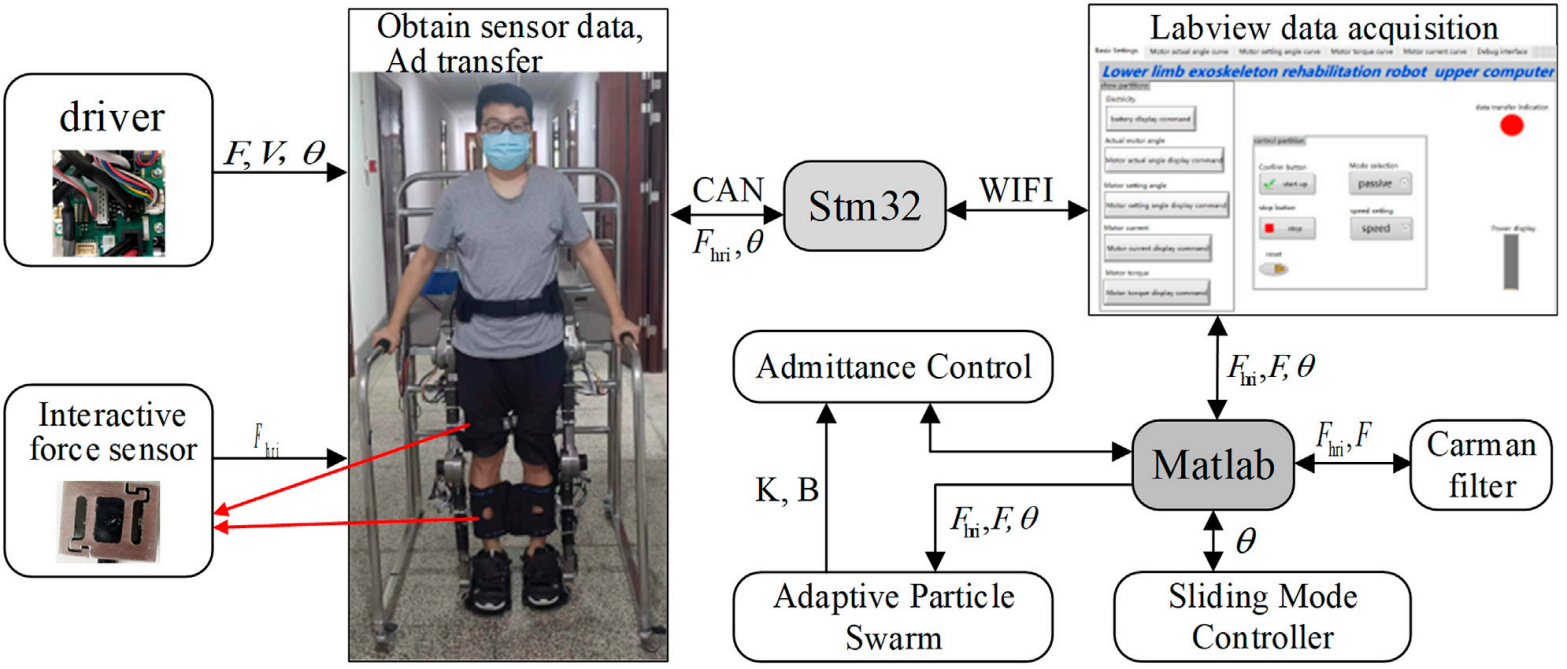
Exoskeleton hardware control platform.

It can be seen from Figure 2 that the sensor system of the lower limb exoskeleton robot contains four interactive force sensors, currents of four joint motors and encoders, that are passed to the direct memory (DMA) of the Stm32 controller through the AD and CAN buses, respectively. Four 2D force sensors are installed at the locations indicated by the red lines in Figure 2, with a range of 0–30 kg and a voltage output range of 0–10 V, which can directly detect the interactive force signals applied to the exoskeleton in the sagittal plane by the thigh and calf. Each joint motor is equipped with a 19-bit absolute encoder having an accuracy of 0.0007°. The Stm32 communicates with Labview on a PC through a WIFI module to perform multi-source sensor information acquisition, and each data curve is displayed in real-time in the interface. In addition, Labview calls the Matlab program to perform signal filtering and calculation of the control algorithm. The training modes of the exoskeleton such as passive training, active training, sitting, and standing modes can be set through the upper computer. The walking speed can also be set in the passive walking mode, which is divided into slow, medium, and fast.

### 2.3 Dynamics model

The 2-degree-of-freedom lower limb exoskeleton dynamics model can be described as:

$$\tau +\tau_{\text{hri }}=M\left(\theta \right)\ddot{\theta }+C\left(\theta \right)\dot{\theta }+G\left(\theta \right)+\tau_{f}+\tau_{d}$$ (1)

where *θ* is the joint motion angle, $\dot{\theta }$ represents the angular velocity, $\ddot{\theta }$ denotes the angular acceleration of the motion, and *τ* represents the input torque of the joint, *τ*_*hri*_ is the introduced human-robot interaction force vector, *τ*_*f*_ is the friction force vector, and *τ*_*d*_ represents the external disturbance vector. Moreover, $M\left(\theta \right)$ represents the inertia matrix, *C*(*θ*) is the centrifugal force and the Coriolis force matrix, and *G*(*θ*) denotes the gravity matrix, as shown in Eq. 1.

## 3 Control solutions

### 3.1 Admittance control

The active rehabilitation training can enhance the motivation of patients to train, which requires a flexible interaction between humans and robots. Admittance control can use the force sensor information to identify predefined kinetic responses. The desired gait position of the human body can be assisted and followed by motors to achieve flexibility. In the admittance model, the kinetic relationship between the desired position of the human body and the human-robot interaction force can be expressed as:

$$f_{\text{ext}}=M_{d}\left(\ddot{\theta }-{\ddot{\theta }}_{d}\right)+B_{d}\left(\dot{\theta }-{\dot{\theta }}_{d}\right)+K_{d}\left(\theta -\theta_{d}\right)$$ (2)

where, *M*_*d*_, *B*_*d*_ and *K*_*d*_ are respectively the inertia, damping, and stiffness matrices, *θ* is the joint motion angle matrix of the human body, *θ*_*d*_ is the desired angle matrix of the robot motion, and *f*_*ext*_ is the human-robot interaction force matrix measured by the force sensor. Note that in this study, the effect of the inertia matrix is ignored due to the small acceleration, such that *M*_*d*_ = 0. The calculation of the desired human gait is performed by collecting the information from the Human-Robot Interaction (HRI) force sensor and the exoskeleton joint angle. It is further derived from Eq. 2 as:

$${\dot{\theta }}_{d}=\dot{\theta }-B_{d}^{-1}\left(f_{\text{ext}}-K_{d}\left(\theta -\theta_{d}\right)\right)$$ (3)

### 3.2 Improved adaptive particle swarm

**FIGURE 3:**
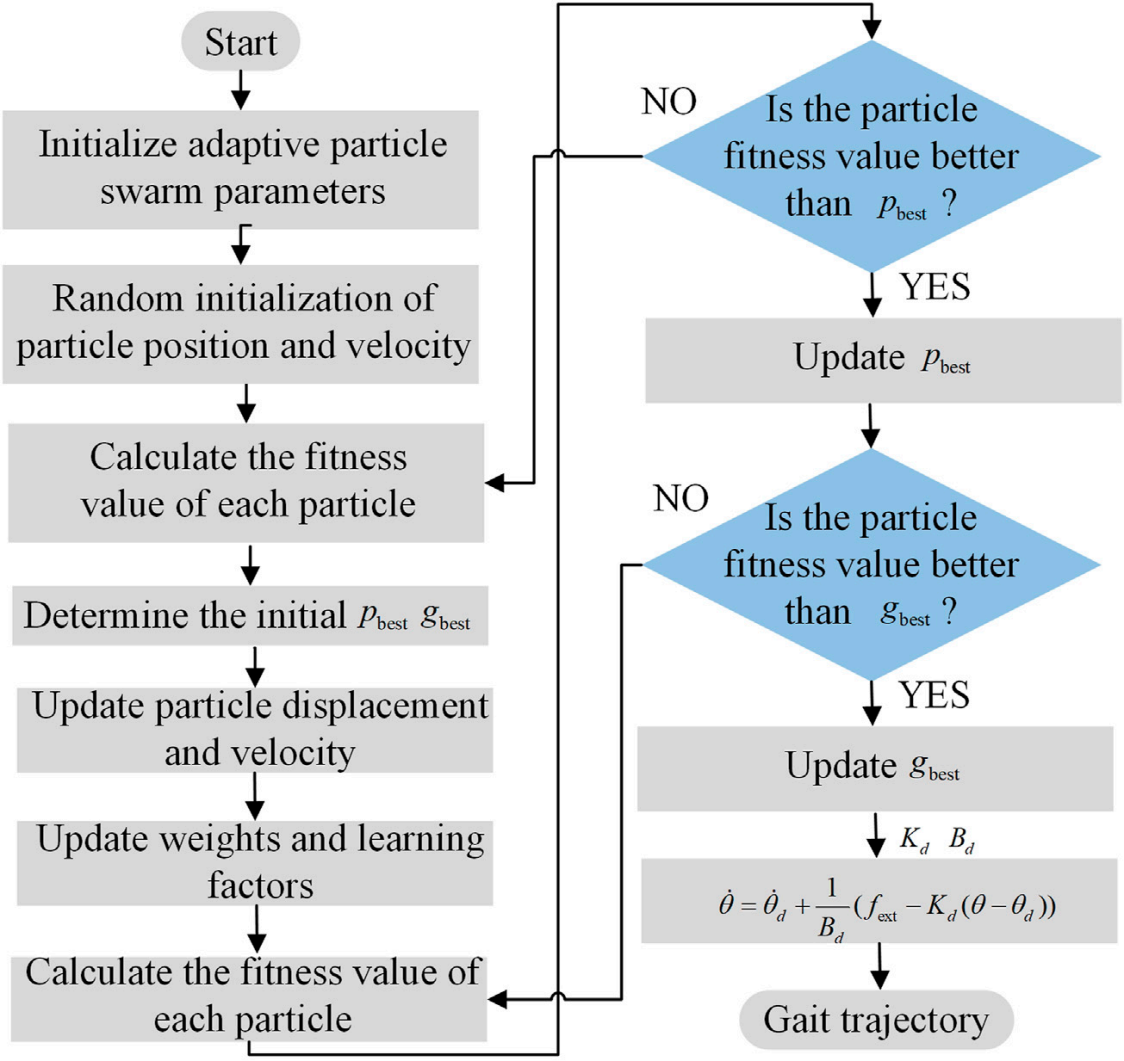
Flowchart of the APSOAC algorithm.

In the walking process, the human gait trajectory has nonlinear characteristics and the speed is constantly changing. Therefore, the fixed parameters of the admittance control cannot adapt to the complex walking process. The conduction parameters, K and B, should be adapted to the walking characteristics of different people and different walking environments. By designing a suitable adaptation function, the online adjustment of the admittance parameters can allow the patients to efficiently perform rehabilitation training with minimal energy consumption and more accurate gait intention tracking. APSO is a population algorithm that simultaneously adapts to multiple targets. Compared with the traditional particle swarm optimization (PSO) algorithm, APSO can adaptively adjust the inertia weights and learning factors which allows the particle population to enhance the global and local search abilities, thus enhancing the adaptability to multi-peaked problems. The use of nonlinear dynamic inertia weights allows them to decrease with the increase of the number of iterations, and to increase with the increase of the distance from the global optimal point, which enhances the global and local search ability of the particles. In the early stage of the algorithm, the learning factor *c*_1_ of the particle itself is large. It relies on the particle itself to maximize the global search. In the later stage of the algorithm, the value of *c*_2_ is larger, and it relies more on the group search to converge to the global optimum. The steps of the APSOAC algorithm are shown in Figure 3. The fitness function of APSO is the performance evaluation function of human-robot interaction, which is expressed as:

$$J=\int_{0}^{+\infty }\left({\left(\theta -\theta_{\text{d}}\right)}^{\text{T}}Q\left(\theta -\theta_{\text{d}}\right)+f_{\text{ext}}^{\text{T}}{Rf}_{\text{ext }}\right)dt$$ (4)

where Q and R are the positive diagonal weight matrices of the trajectory tracking error and the human-robot interaction force, respectively. Following Eq. 10, the generated gait trajectory is considered as the optimal trajectory of the robot under the action of external forces.

The particle displacement and velocity are updated as follows:

$$x_{i,j}\left(t+1\right)=x_{i,j}\left(t\right)+v_{i,j}\left(t+1\right),\;j=1,\ldots ,n$$ (5)

$$v_{i,j}\left(t+1\right)=\omega \times v_{i,j}\left(t\right)+c_{1}r_{1}\left[p_{i,j}-x_{i,j}\left(t\right)\right]+c_{2}r_{2}\left[p_{g,j}-x_{i,j}\left(t\right)\right]$$ (6)

**FIGURE 4:**
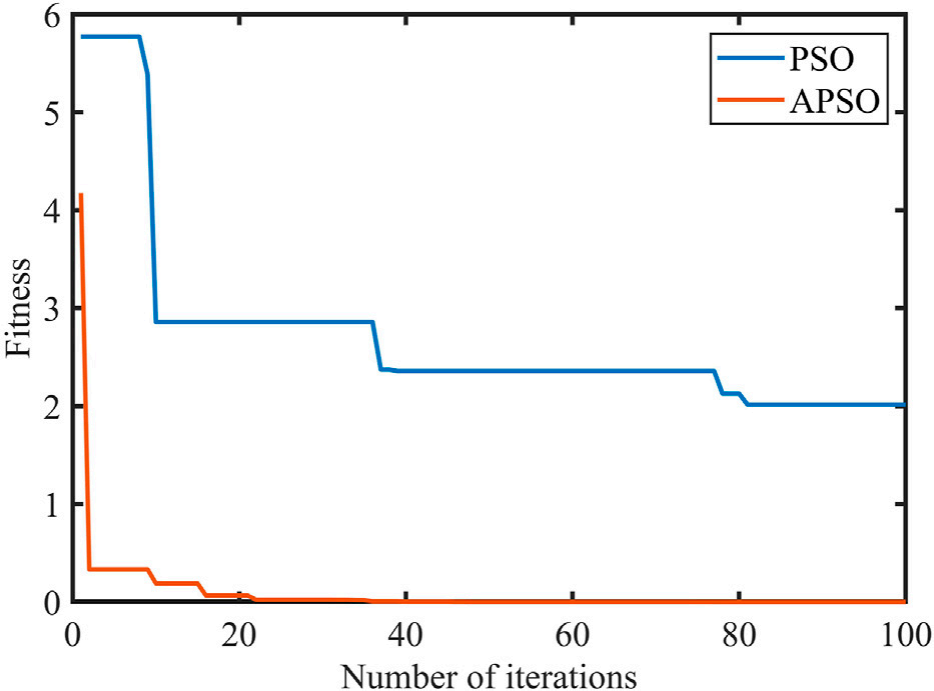
The fitness function of two optimization algorithms.

The weights and learning factors are then updated using Eqs 7–9.

$$\omega =\left\{\begin{array}{cc} \omega_{\min}-\frac{\left(\omega_{\max}-\omega_{\min}\right)\times \left(f-f_{\min}\right)}{f_{\text{avg }}-f_{\min}},\; & f\leq f_{\text{avg }}\\ \omega_{\max},\; & f>f_{\text{avg }} \end{array}\right.$$ (7)

$$c_{1}=2e^{-j/N}$$ (8)

$$c_{2}=\left(2/e\right)*e^{\text{j}/N}$$ (9)

where N is the number of particles in the population, *ω*_max_ and *ω*_min_ are the maximum and minimum values of the inertia weights, respectively, *f* denote the real-time objective function values of the particles, *f*_*avg*_ and *f*_min_ represents the average and minimum objective values of all the current particles, respectively, is the number of current iterations, *c*_1_ and *c*_2_ denote the learning factors of the particles. The judgment statement of the flowchart compares the adaptation value of each particle with its best position. If they are the same, the current value is used as the best position of the particle. All the current *p*_*best*_ and *g*_*best*_ are then updated. When the algorithm reaches its stopping condition, it stops the search and outputs the admittance control parameters K and B for both hips. If the stopping condition is not satisfied, it returns to step 3. The simulation curves of the fitness functions of APSO and PSO are shown in Figure 4, which demonstrates the variation of the fitness with the increase in the number of iterations. It can be seen that the performance of the improved APSO outperforms the traditional PSO algorithm. The improved APSO algorithm converges faster and is able to jump out of the local optimal solution continuously during the iterative process and reaches the optimal solution, thus achieving the optimal human-computer interaction effect.

### 3.3 RBF neural network adaptive sliding mode control

When rehabilitation training is performed on the ground, the control system faces many challenges, such as frictional forces, interaction forces between the ground and the exoskeleton footbed, and uncertainty of external perturbations. The radial basis function neural networks have high approximation performance for any nonlinear system as well as high resistance to noise. Therefore, radial basis functions are used to approximate the frictional forces and perturbations in the exoskeleton human-robot system. RBF neural networks are fused with sliding mode control, and RBF neural network adaptive sliding mode control is proposed to track the gait profile planned by adaptive particle swarm admittance control (APSOAC). In general, the RBF neural network can be represented by:

$$h_{j}=\exp\left(\frac{{\left\|\theta -c_{j}\right\|}^{2}}{2b_{j}^{2}}\right)$$ (10)

$$\tau_{f}\left(\theta \right)=W^{*\;T}h_{f}\left(\theta \right)+\varepsilon_{f}$$ (11)

$$\tau_{d}\left(\theta \right)=V^{*\;T}h_{d}\left(\theta \right)+\varepsilon_{d}$$ (12)

where *θ* is the input of the neural network, *h* is the output of the Gaussian basis function, W* and V* are respectively the ideal weights of *τ*_*f*_ and *τ*_*d*_, *ɛ*_*f*_ and *ɛ*_*d*_ are the approximation errors of the network, *j* represents the *j*th node of the network hidden layer, *c* is the coordinate vector of the centroid of the neuron Gaussian basis function in the hidden layer, and *b* is the width of the neuron Gaussian basis function in the hidden layer. Taking *τ*_*f*_ as an example, the weights of the RBF network are obtained using the gradient descent method. In order to prevent overfitting, we also adopt the dropout regularization technique to enhance the model’s generalization ability. The error of the network approximation is evaluated using an error indicator.

$$E\left(\theta \right)=\frac{1}{2}{\left[\tau_{\text{hri}}\left(\theta \right)-W_{j}^{*}\left(\frac{{\left\|x-c_{j}\right\|}^{2}}{2b_{j}^{2}}\right)\right]}^{2}$$ (13)

To minimize the error indicator function, the weights of the network are adjusted using the gradient descent method.

$$\Delta W_{j}\left(t\right)=-\eta \left[\tau_{\text{hri}}\left(\theta \right)-{\hat{\tau }}_{\text{hri}}\left(\theta \right)\right]h_{j}$$ (14)

$${\hat{\tau }}_{f}\left(\theta \right)=M\left({\hat{W}}^{\text{T}}h_{f}\left(\theta \right)\right)$$ (15)

$${\hat{\tau }}_{d}\left(\theta \right)=M\left({\hat{V}}^{*\;T}h_{d}\left(\theta \right)\right)$$ (16)

where $\hat{W}$ and $\hat{V}$ are the estimated weights. $\tilde{W}=\hat{W}-W^{*}$ , $\tilde{V}=\hat{V}-V^{*}$ . The ideal angle *θ*_*d*_, error *e* = *θ*_*d*_ − *θ*, and sliding mode function are expressed as:

$$s=ce+\dot{e},\;c>0$$ (17)

where c represents the gain parameter of the sliding mode controller. The design control law is given by:

$$\tau +\tau_{\text{hri }}=M\left(-ce+{\ddot{\theta }}_{d}-\eta \mathrm{sgn}\left(s\right)\right)+C\left(\theta ,\dot{\theta }\right)\dot{\theta }+G\left(\theta \right)+{\hat{\tau }}_{f}+{\hat{\tau }}_{d}$$ (18)

where *η* is the slope parameter of the sliding surface. The Lyapunov function is chosen as:

$$L=\frac{1}{2}s^{2}+\frac{1}{2\gamma_{1}}{\tilde{W}}^{\text{T}}\tilde{W}+\frac{1}{2\gamma_{2}}{\tilde{V}}^{\text{T}}\tilde{V}$$ (19)

*γ*_1_ and *γ*_2_ are commonly referred to as regularization parameters.

$$\begin{array}{cc} \dot{L} & =s\dot{s}+\frac{1}{\gamma }{\tilde{W}}^{\text{T}}\dot{\hat{W}}+\frac{1}{\gamma_{2}}{\tilde{V}}^{\text{T}}\dot{\hat{V}}\\ & =s\left\{c\dot{e}+M^{-1}\left[\tau +\tau_{\text{hri}}-C\left(\theta \right)\dot{\theta }-G\left(\theta \right)-\tau_{f}-\tau_{d}\right]-{\ddot{\theta }}_{\text{d}}\right\}+\frac{1}{\gamma }{\tilde{W}}^{\text{T}}\dot{\hat{W}}+\frac{1}{\gamma_{2}}{\tilde{V}}^{\text{T}}\dot{\hat{V}}\\ & ={\tilde{W}}^{\text{T}}\left[sh_{f}\left(\theta \right)+\frac{1}{\gamma_{1}}\dot{\hat{W}}\right]+{\tilde{V}}^{\text{T}}\left[sh_{d}\left(\theta \right)+\frac{1}{\gamma_{2}}\dot{\hat{V}}\right]+s\left[-\varepsilon_{f}-\eta \mathrm{sgn}\left(s\right)-\varepsilon_{d}\right] \end{array}$$ (20)

The adaptive law is considered as:

$$\dot{\hat{W}}=-\gamma_{1}sh_{d}\left(\theta \right)$$ (21)

$$\dot{\hat{V}}=-\gamma_{2}Sh_{d}\left(\theta \right)$$ (22)

Therefore:

$$\dot{L}=s\left[-\varepsilon_{f}-\eta \mathrm{sgn}\left(s\right)-\varepsilon_{d}\right]$$ (23)

**FIGURE 5:**
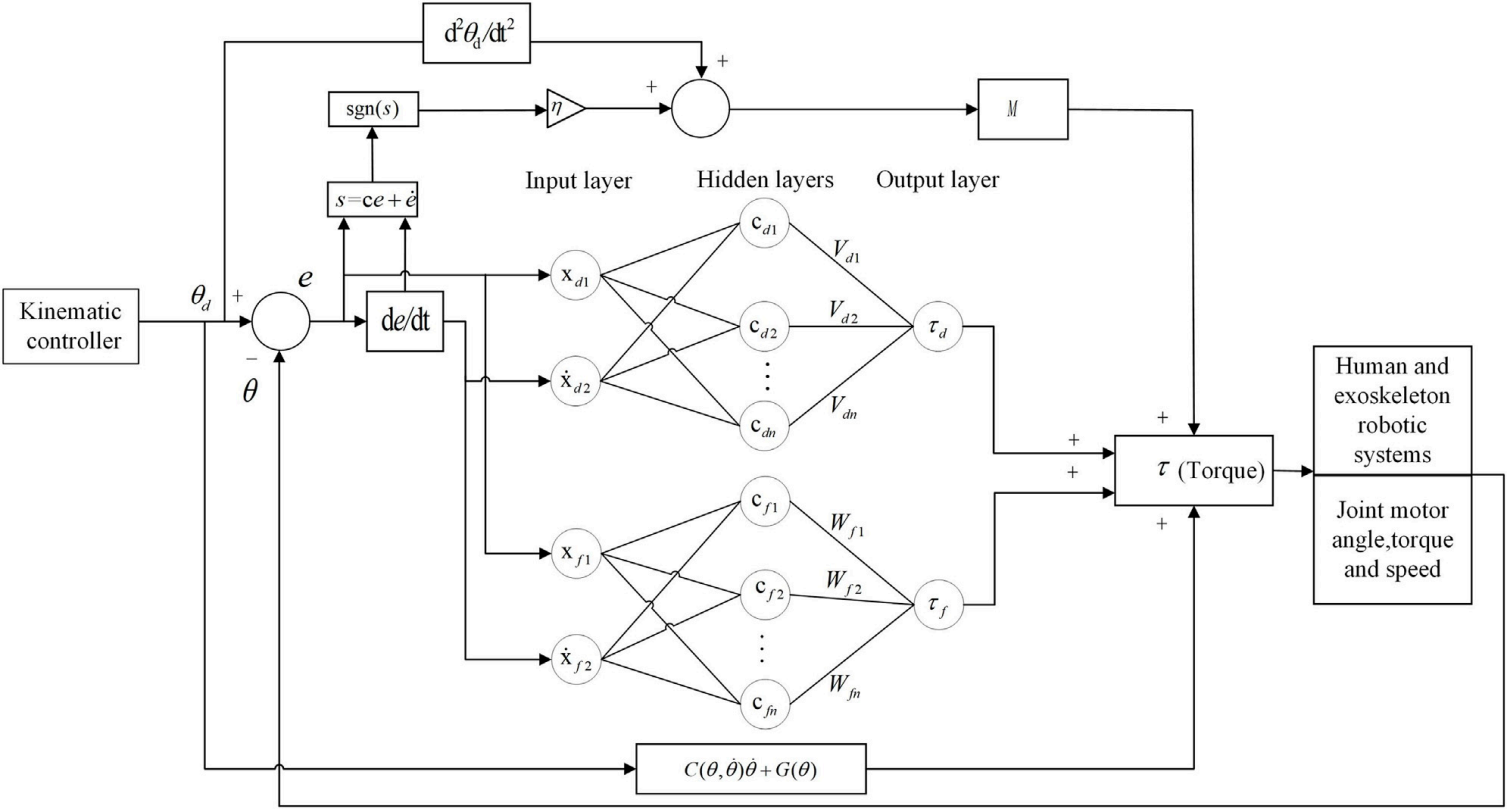
RBF dual adaptive sliding mode control system for lower limb exoskeleton robot.

The approximation errors *ɛ*_*f*_ and *ɛ*_*d*_ of the RBF network are very small real numbers. Take *η* greater than or equal to the absolute value of tne sum of *ɛ*_*f*_ and *ɛ*_*d*_. Then $\dot{L}\leq 0$ . When $\dot{L}\equiv 0$ , *s* ≡ 0, according to the LaSalle invariant set principle, and when *t* → *∞*, *s* → 0. The dual RBF adaptive sliding mode control is shown in Figure 5. The sliding mode control acts on the inertia matrix of the exoskeleton dynamics, and the two radial basis functions are used to fit the friction and external disturbances in the exoskeleton dynamics model. The encoder in the exoskeleton motor is used to measure the control system error and the rate of error change as the input of the RBF network. *ɛ*_*f*_ and *ɛ*_*d*_ are the outputs of the two RBF functions. The adaptive law is designed to keep the control system stable, which can compensate for the approximation error of the RBF neural network in order to improve the system performance. Finally, the proof is carried out by the Lyapunov stability theory.

## 4 Experiment

### 4.1 Introduction

Three healthy subjects (two males and one female, age 27 ± 3 years) were first trained in active and passive walking rehabilitation. Note that this study was approved by the Ethics Committee of the School of Mechatronics of Beijing University of Technology, and all the procedures were carried out according to the standards of the Declaration of Helsinki. The experiment was divided into two main parts: active walking and passive walking. To evaluate the trajectory tracking the effect of DRNNASMC, the passive walking experiment consists of four parts: sitting and standing, slow walking, medium speed walking, and fast walking. The active walking experiment consists in evaluating the softness of APSOAC’s control and the performance of human-robot interaction.

### 4.2 Active walking experiment

In the active walking experiment, the motion trends at the hip and knee joints of both legs were sensed by four force sensors. A subject was asked to walk twice using the same step frequency and stride length according to two control schemes: APSOAC and admittance control (AC). The walking training effect of the two control schemes was then judged by comparing the profiles of HRI force and joint motor current in the two experiments. Due to the periodic nature of the HRI force signal and the noise effect, the root mean square (RMS) value was used to quantify the HRI force and motor-assisted force (expressed as current) (Zhuang et al., 2020):

$$RMS=\sqrt{\frac{1}{T}\overset{T}{\underset{i=1}{\sum }}{\left(y\left(i\right)\right)}^{2}}$$ (24)

**FIGURE 6:**
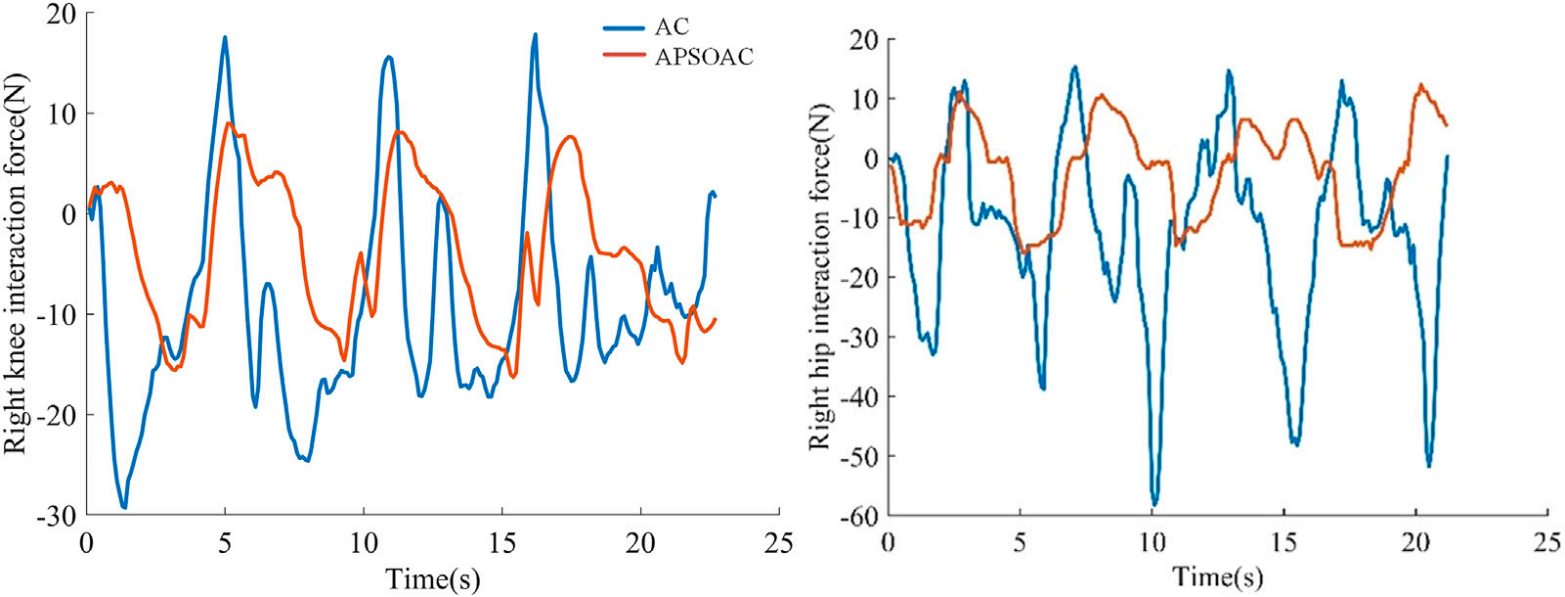
Human-robot interaction forces in the hip and knee joints.

The orange and blue lines in Figure 6 show the human-exoskeleton interaction forces for the APSOAC and AC control schemes, respectively. The human-robot interaction force curve for the APSOAC algorithm varies between −15 N and 10 N. It is then in the tolerable interaction force range. The blue line indicates the interaction force curve of the AC algorithm, which varies between −60 N and 20 N. The interaction force fluctuates more, the human body is subjected to high resistance, and the human-robot interaction is unstable. Based on the values presented in the root mean square values of the hip and knee joint interaction forces, it can be observed that the effective interaction force on the thigh and calf are relatively small. The *τ*_hri,hip_ and *τ*_hri,knee_ of APSOAC are 8.42 N and 8.28 N, which are lower than the 20.10 N and 13.83 N of AC, respectively. This indicates that the APSOAC scheme results in a smaller human-robot interaction force, with less variability in the force, ultimately leading to better human-robot interaction performance.

**FIGURE 7:**
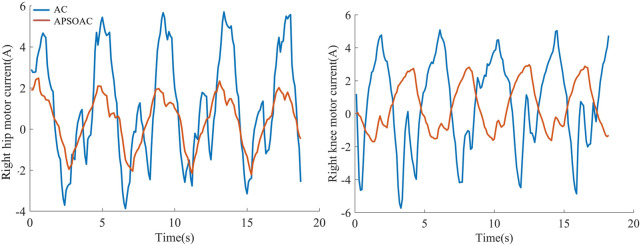
Current in the hip and knee joints.

The current loops of the hip and knee motors were used as a reference for the motor-assisted force. The root-mean-square values of the motor currents are shown in Figure 7. Under the same walking conditions, the auxiliary force of AC is significantly larger than that of APSOAC. The variation interval of the current of APSOAC is [-2 A, 3 A] with a more stable current variation, while the AC control scheme requires to overcome the larger inertia brought by the gait phase change, such as the area around 5, 10, and 15 s, due to the fixed parameters. Based on the values presented in the root mean square values of currents in hip and knee motors, it can be observed that the auxiliary currents of the motor to the human hip and knee joints in the APSOAC scheme are respectively 1.3 A and 1.51 A. They are 44.83% and 53.55% of the auxiliary currents of the AC control scheme. This also illustrates that the conventional admittance control with fixed parameters cannot adapt to the complex gait variations.

**FIGURE 8:**
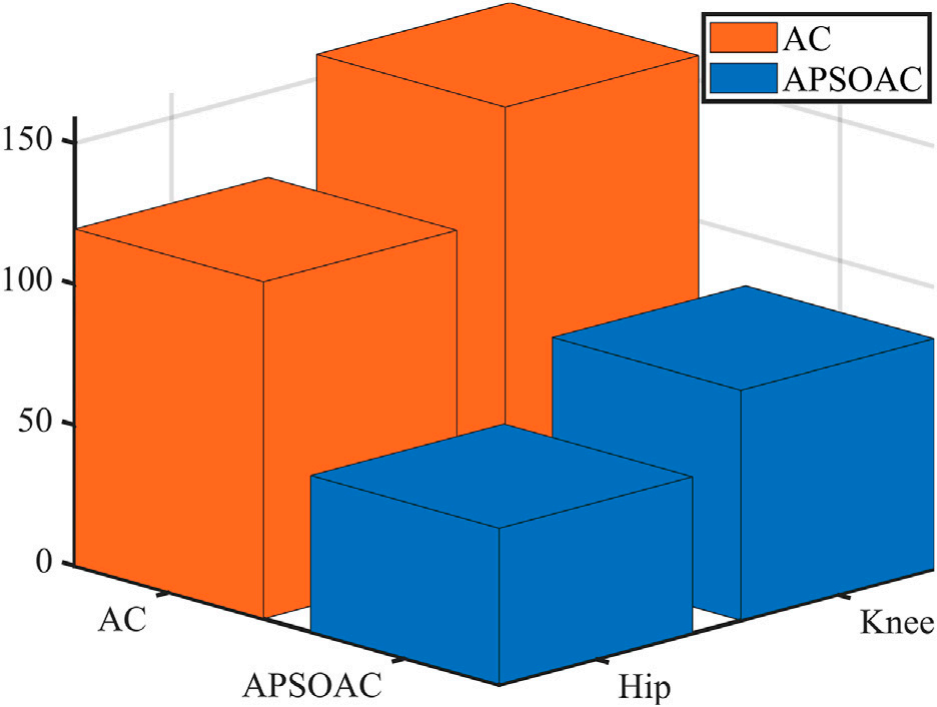
Energy consumed in the hip and knee joints for the two control schemes.

Figure 8 shows the difference between the two control schemes in terms of single-step energy consumption, that is, the electrical energy consumed by the subjects for each step. The average electrical energy consumed by the AC algorithm for the hip and knee joints in a single-step walk was respectively 119.571 and 158.744 J, while that consumed by the APSOAC algorithm was respectively 55.547 and 81.6802 J. The results indicate that, compared with the conventional admittance algorithm, the APSOAC algorithm can save 53.5% and 48.5% of energy for the hip and knee joints when walking, respectively. It can also save 50.7% of electrical energy on average. The electrical energy saving also indicates that the human body’s physical energy is saved accordingly, and the training is smoother and more efficient.

### 4.3 Passive walking experiment

Passive walking experiments were conducted to evaluate the trajectory control effects of the exoskeleton robot in sitting, standing, and walking at different speeds by quantitative analysis. The root-mean-square values of the position tracking error were analyzed by completing a given gait training trajectory. The sitting and standing experiments are equivalent to step trajectory following, where the exoskeleton is adjusted to sitting mode before the subject puts on the exoskeleton. The human body then puts on the exoskeleton and sets it to standing mode, so that the subject changes from sitting to standing and the walking training can be performed. The steep curve of seated standing is used to analyze the error between the actual motion and the target trajectory, so as to study the influence of the robot’s following motion on a single target position. In the slow, medium, and fast walking training, the subjects wear exoskeletons and follow the gait curves at different preset gait speeds, in order to analyze the trajectory tracking effects of the DRNNASMC and conventional PID algorithms at different gait speeds.

#### 4.3.1 Sitting and standing tracking experiments

**FIGURE 9:**
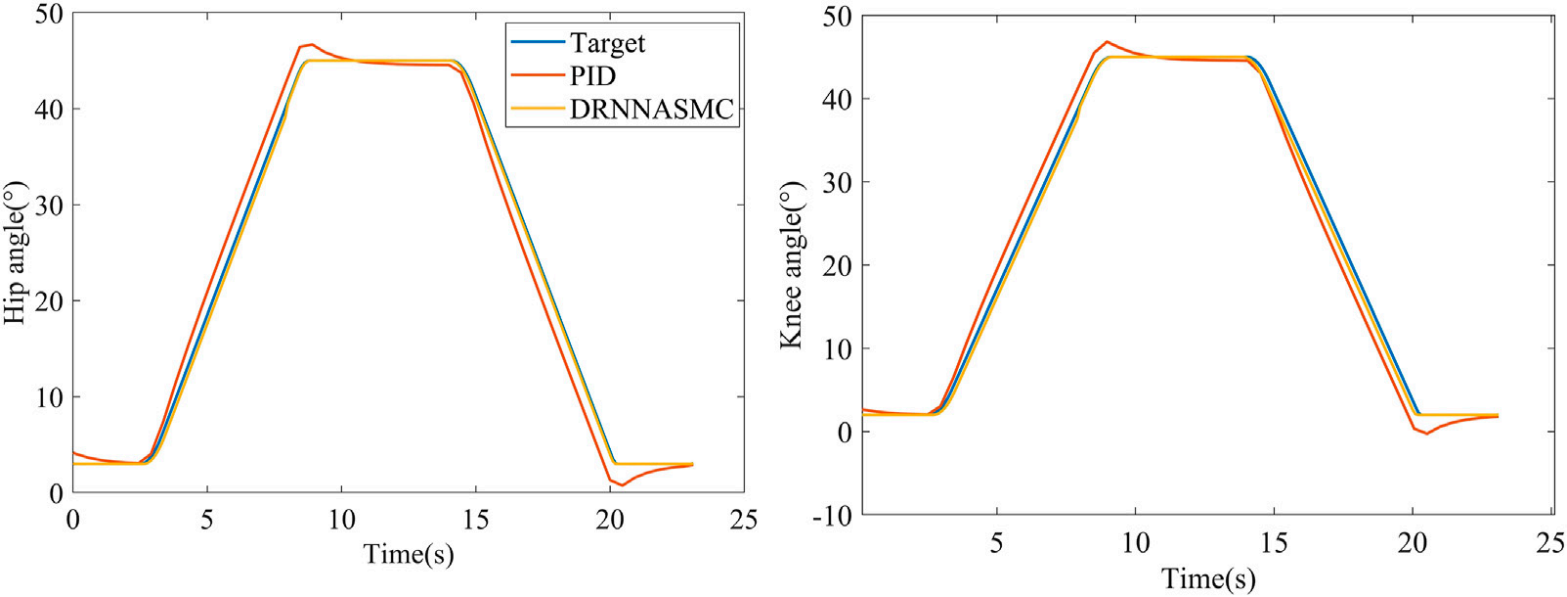
Tracking curves of two control schemes in sitting and standing modes.

In Figure 9, the curve segment from 3 to 8 s is the process of the exoskeleton changing from standing to sitting, while the curve segment from 15 to 20 s is the process of changing from sitting to standing. At 8 and 20 s, when the motor reaches the target angle, the PID controller does not stop the rotation immediately, but overshoots. However, the curve of the DRNNASMC controller coincides with the reference trajectory while having less errors.

**FIGURE 10:**
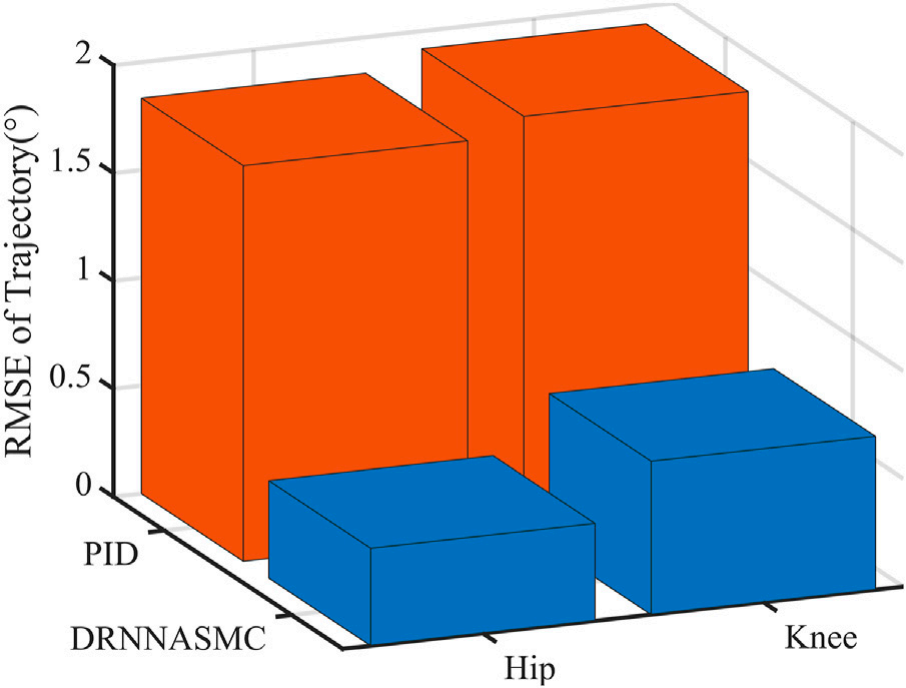
Root mean square error of two control schemes in sitting and standing modes.

The root-mean-square values of the trajectory tracking errors of DRNNASMC and PID are shown in Figure 10. The root-mean-square errors of the PID algorithm on the hip and knee joints are respectively 1.833° and 1.918°, and those of the DRNNASMC algorithm are 0.452° and 0.711°, respectively. The results show that the tracking errors of the DRNNASMC algorithm are significantly smaller than those of the PID algorithm, which indicates that the control scheme has a significant effect on the tracking error.

#### 4.3.2 Passive walking trajectory tracking experiments with different step speeds

**FIGURE 11:**
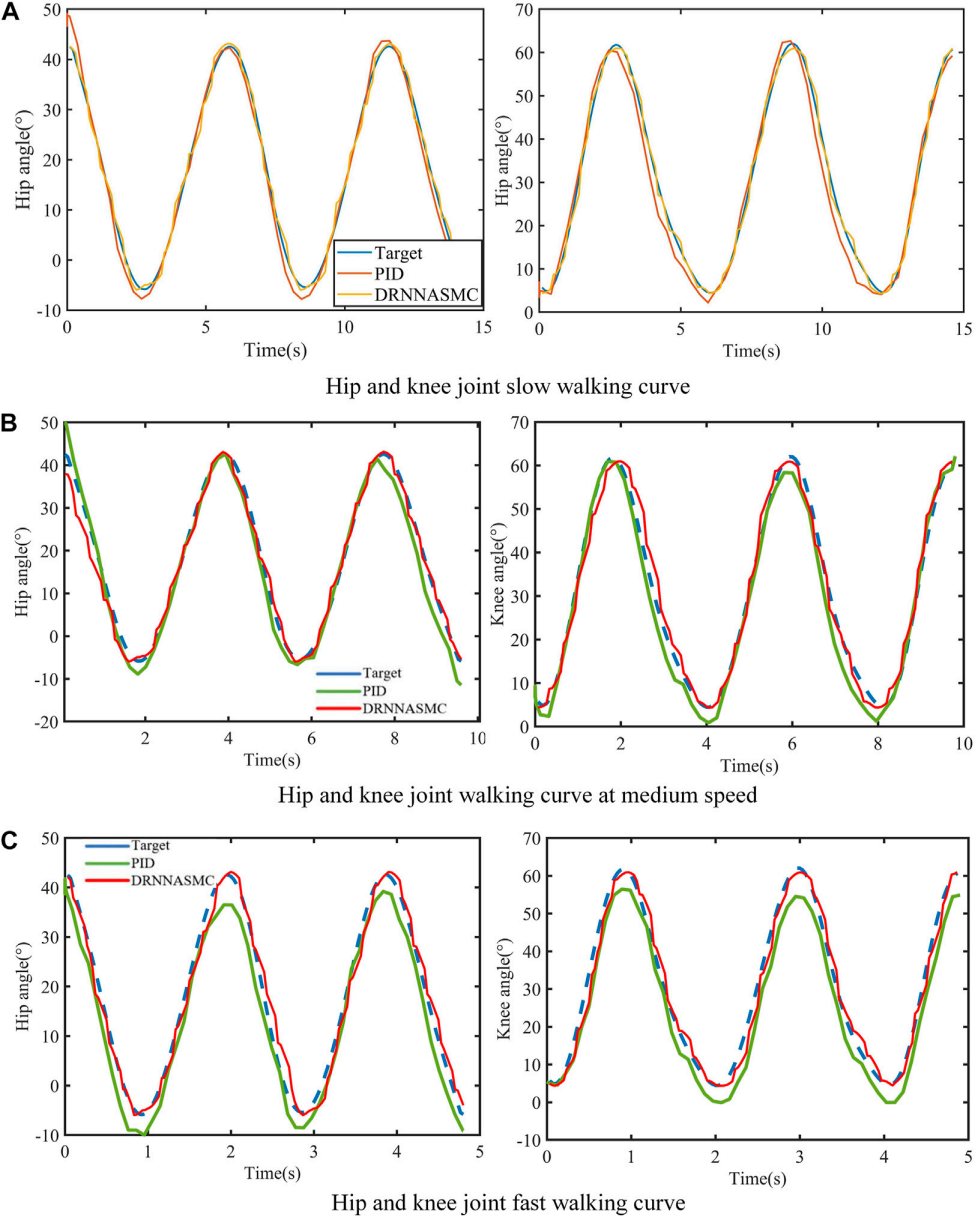
Walking gait curves at three walking speeds. **(A)** Hip and knee joint slow walking curve. **(B)** Hip and knee joint walking curve at medium speed. **(C)** Hip and knee joint fast walking curve.

Figure 11 shows the tracking performance of the hip and knee joints for both control schemes over two half-cycles. By qualitatively analyzing the tracking curves, it can be deduced that the tracking error of the PID control curve is increasing with the increase of the gait training speed, and the error mainly appears in the gait change phase (at the crest and trough of the figure). The DRNNASMC control curve is less affected by the speed increase, and the tracking accuracy is higher.

**FIGURE 12:**
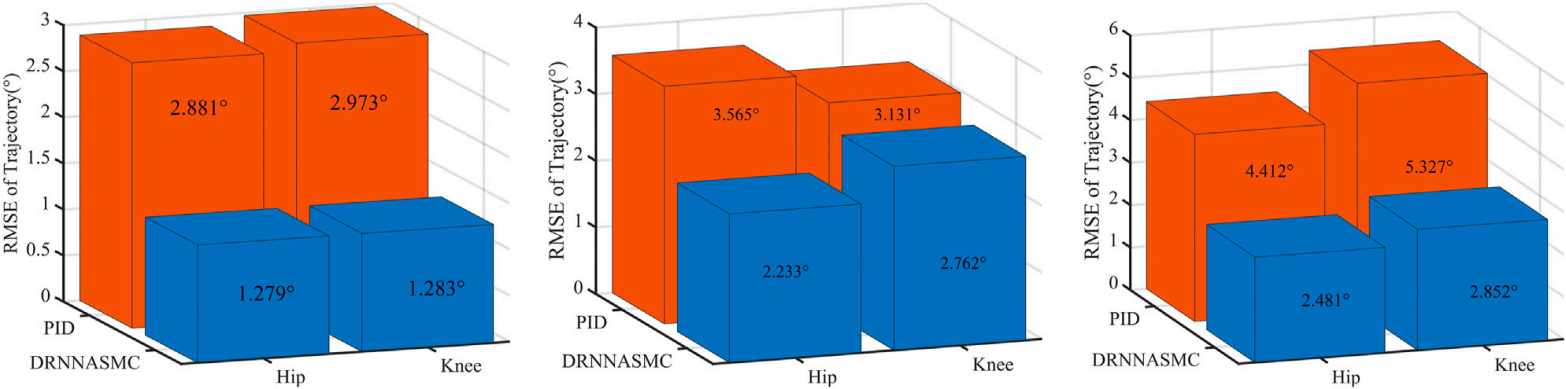
Root mean square error for two control schemes in slow, medium, and fast travel modes.

The RMSE values of the hip and knee tracking data from the two control schemes were then quantified (Figure 12). The root means square error results show that the control scheme significantly affects the gait trajectory tracking efficiency. When the walking speed increased, the tracking error of the PID controller increased, with a larger tracking bias. The tracking error of the DRNNASMC controller increased from low to medium speed. However, there was no significant error difference from medium to high speed. In addition, the average error of DRNNASMC over the hip and knee joints was 56% smaller than that of the PID controller in each speed level from low to high speed (25% and 45%, respectively).

#### 4.3.3 Active walking trajectory tracking experiment

**FIGURE 13:**
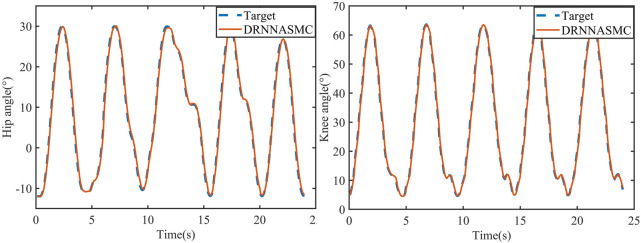
DRNNASMC algorithm tracking curve.

Gait curves were generated using APSOAC, and trajectory tracking was performed using the DRNNASMC controller, as shown in Figure 13. It can be seen that compared with the gait curves during passive training, the gait tracking for active training was better and the curves were smoother due to less human-robot interaction forces. It can be observed from the histogram that the RMSE values for hip and knee tracking errors are 0.88° and 0.86°, respectively. These results are significantly lower than the gait-following errors for passive training. This further demonstrates that APSOAC accurately identifies the human motion intention, and it is able to move in the direction where the human-robot interaction force is reduced, making the active walking more supple, thus achieving a good tracking effect.

## 5 Discussion

It can be seen from the experimental results of active walking training (Figure 6) that the human-robot interaction force for the hip and knee using the proposed APSOAC algorithm is respectively 58.1% and 40.1% smaller than that of the AC algorithm. It can be observed from the current curves of each joint (Figure 7) that the experimental results are similar to the human-robot interaction force curve (Figure 6), which indicates that the motor provides less auxiliary force than that of the AC controller when the same target position is reached during human walking with APSOAC control. In addition, it can be deduced from the single-step energy consumption in Figure 9 that the active training using the APSOAC algorithm saves an average of 50.7% of electrical energy, compared with the admittance control. The experimental results in Figure 6 and Supplementary Figure S8 show that when the human leg steps forward, the APSOAC controller can accurately identify its motion trend in real-time. In addition, the adaptive particle swarm allows the admittance controller to derive the desired motion trajectory of the human body, by updating the optimal K and B in real-time. While the adaptive particle swarm algorithm offers several advantages over traditional AC algorithms, it may still be susceptible to local optima. Future research will focus on studying and improving this limitation. The DRNNASMC controller can then make the motor continuously move in the direction of the human-robot interaction force reduction. Thus, the human body’s desired position is achieved by the DRNNASMC controller.

**FIGURE 14:**
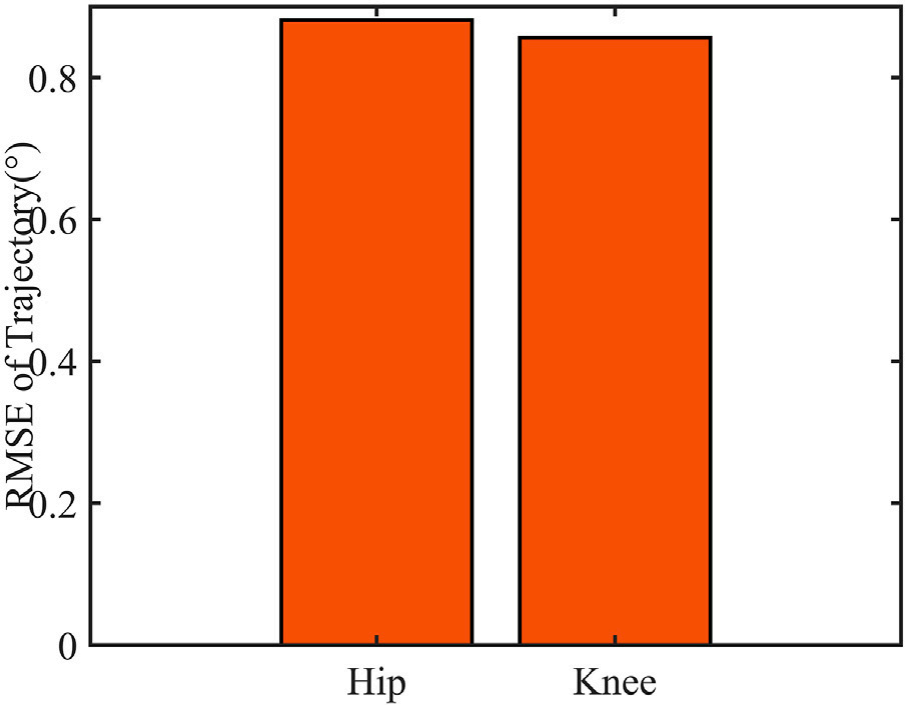
Root mean square error of the two control schemes in active walking mode.

It can be seen from Figures 12, 14 that the accuracy of active walking on gait curve tracking is higher than that of passive walking. This is due to the fact that the human leg does not exert force when passively walking, they rely on the joint motor for driving, and the motor resistance is larger. In active walking, the human leg is active and the exoskeleton follows its movement. Therefore, the human-robot interaction force is small. The dual RBF network based on the dynamics model quickly compensates the torque required by the motor. Thus, the error of gait curve tracking for the hip and knee joints is small, and the RMSE values are 0.88° and 0.86°, respectively. The latter values are 31.2% and 33% lower than the tracking error in passive slow walking. The results show that the APSOAC algorithm provides accurate human motion intent and performs supple control in active walking training. In the passive walking experiment, the error of the DRNNASMC controller increases with the speed increase, fluctuating within 2.9, with an error fluctuation range 46.5% smaller than that of the PID controller. This also demonstrates the efficiency of the proposed DRNNASMC algorithm and its relative insensitivity to external perturbations.

## 6 Conclusion

The proposed paper introduces a lower limb exoskeleton robot that utilizes an adaptive particle swarm-optimized conductance control algorithm to achieve gait trajectory generation. This algorithm enhances the human-robot interaction, making it more flexible, and enables the planning of desired human body gaits. By incorporating the adaptive particle swarm algorithm, the gait planning becomes adaptable. Additionally, a dual RBF neural network adaptive sliding mode controller is employed to compensate for external perturbations and friction. This controller ensures accurate tracking of the gait planning curve by the lower limb exoskeleton. Finally, experimental demonstrations validate the aforementioned studies. Overall, these studies offer a more effective and sustainable rehabilitation research program for stroke patients’ rehabilitation.

## Data Availability

The raw data supporting the conclusion of this article will be made available by the authors, without undue reservation.
